# Catalogue of Such British Plants as Have Been Found in Any Shape Serviceable to Man, Whether in a Medicinal, Œconomial, Culinary, or Agricultural Point of View, Together with an Account of the Uses Which They Have Been Made to Anwser, and an Accurate Botanical Description of Each Plant

**Published:** 1804-07-01

**Authors:** 


*Catalogue of such British Plants as have been found in any Shape serviceable to Man, whether in a medicinal, œconomial, culinary, or agricultural Point of View, together with an Account of the Uses which they have been made to anwser, and an accurate Botanical Description of each Plant.*

[ Continued from Vol. xi. pp. 526—534. ]

8. Rubia. *R. tinctorum.*—*R. peregrina.* *R. Sylvestris aspera.* *R. T. Anglica.*

*Ang.* Madder, Dyer's madder, Wild madder.

*Gen. Desc.* Bloss. 1-petal; bell shaped; berry 2-seeded.

*Spec. Desc.* *Leaves* annual; *stem* prickly, 4-cornered, climbing. *Bloss.* dirty yellow. The *variety* of this plant, which is a native of England, having the *blossom* 5 *div.*, *leaves smooth, shining, deciduous,* differs in several respects from any of the Linnean species. *See Withering Bot. Arr.* *Hedges in Devonshire; Isle of Wight, &c.* Bloss. June, July.

*Use.* Madder has been considered as *diuretic,* (which is

 denied denied by Dr. Cullen) *deobstruent,* and *detergent,* and is chiefly used in the *jaundice* (on the authority of Sydenham), *dropsy* and other diseases supposed to proceed from *visceral obstructions,* particularly those of the liver and kidneys; some modern authors have recommended it as an *emmenagogue* (Home, Clinical Exp.) and in *rickety* affections (Levret) which appears surpising, as brute animals, to which it was given, especially the younger, suffered considerable emaciation and prostration of strength from its effects. Dr. Home gave it as an emmenagogue, from a scruple to half a drachm of the powder, or two ounces of the decoction, three or four times a day. Dr. Cullen says, that "after several ineffectual trials, practitioners have entirely deserted its use." It has the peculiar property of tinging with a florid red colour, the milk, urine, cutaneous excretion, and even the bones of animals that have fed upon it; the bones are quickly affected, and the colouring matter extends through the whole osseous substance, so that if this root be intermitted and employed at proper intervals, the bones are found to be coloured in a correspondent number of concentric circles. Though the cultivation of madder in Britain seems to promise much advantage both to the planter and the nation, yet the great quantities of those roots used by the British dyers and callico-printers, has been for many years almost wholly the growth and export of Holland. But lately, by the laudable exertions of the *Soc. for the Encouragement of Arts, &c.* considerable quantities of English madder have been produced, and found as good at least, if not better than any imported. *See Transac. v.* 1, *p.* 10.—*Woodville.* For a full account of the cultivation of madder, see *Miller's Dict. in artic.*

9. Plantago. *P. Major*; *P. vulgaris*; *P. latifolia-vulgaris.*

*Ang.* Way-bread, Common great plantain.

*Gen. Desc.* Bloss. 4-cleft, permanent, border broken back. cal. 4-cleft; stam. extremely long; caps. 2-celled, cut round, superior.

*Spec. Desc. Leaves* egg-shaped, smooth with 7 or 9 ribs. *Stalk* cylindrical, nearly 2-edged, 9 to 18 inches high. *Spike* tiled with florets, rather rough with short hairs. *Road sides, very common.* Bloss. June, August.

*Use.* This plant, though omitted in the London, is retained in the Edinburgh Pharmacopœia, in which the leaves are mentioned as the pharmaceutical part of the plant; plant: their qualities are said to be *refrigerant, attenuating, substyptic, and diuretic.* It was formerly reckoned amongst the most efficacious of vulnerary herbs, and by the peasants the green leaves are now commonly applied to cuts and other fresh wounds, and cutaneous sores; sometimes made into an ointment. Inwardly, they have been used in phthisical complaints, spitting of blood, and in various fluxes, both alvine and hæmorrhagic. The *seeds*, however, seem better adapted to relieve pulmonary diseases than the leaves, as they are extremely mucilaginous. The *roots* have been recommended for the cure of *tertian interemittents*; and, from the experience of Bergius, not undeservedly. An oz. or two of the expressed juice, or of a strong infusion of plantain, may be given for a dose; in agues, double that quantity, taken at the commencement of the fit.—*Woodville.* Plantain has been alleged to be a cure for the bite of a rattle snake, probably with little foundation, though it is one of the principal ingredients in the remedy of the Negro Cæsar, for which he was rewarded by the Assembly of S. Carolina.—*Duncan's New Edinb. Dispen.* Sheep, goats and swine eat it; horses and cows refuse it.

10. Cornus. *C. Sanguinea; C. fœmina.*

*Ang.* Dogberry tree. Hounds tree. Hounds berry. Prickwood. Prick timber. Gatten tree. Female Dogwood. Femalcornel.

*Gen. Desc.* Involucrum generally 4-leaved; petals 4, superior; drupa succulent, beneath, 2-celled, hard, solitary.

*Spec. Desc.* *Stem* 4 or 5 feet high, dark brown. *Branches* straight; shoots red; *leaves* egg-spear shaped with strong nerves, green on both sides; *tuft of flowers* flatted, in 5 divisions, these subdivided; *flowers* white. *Hedges and woods.* Bloss. June.

*Use.* The berries of this tree have a styptic quality, and are bitter to the taste. They serve also to dye purple. The wood is white and very hard and smooth, fit for the purposes of the turner. The leaves change to a blood red in autumn. Horses, sheep and goats eat it; cows and swine refuse it.

11. Parietaria. *P. officinalis. P. vulgaris. P. Dioscoridis.*

*Ang.* Pellitory of the Wall.

*Gen. Desc.* Female florets mixed with hermaphrodite on on the same branch; calyx 4-cleft; bloss. o; seed, 1, superior, lengthening.

*Spec. Desc. Leaves* spear-egg-shaped; *fruit-stalks* forked; *stems* reddish; *cup* 2-leaved; *bloss.* greenish white; *anthers,* if touched when ripe with a pin, burst, and emit their pollen with some considerable force; sometimes fly from the calyx—*Light. Old walls, rubbish, v. com.* Bloss. May—September

*Use.* This plant was formerly in repute as a medicine; but it does not seem to possess any remarkable qualities: it was accounted an *emollient,* though apparently without reason; and as a *diuretic* its character is better known; its expressed juice, sweetened with sugar, is said to have had a powerful effect in that way; and a decoction of this plant and *uva ursi* has been found of use in clearing the urinary passages. It is now, however, very seldom used, t ough retained in both Pharmacopeias.—*Woodville.* *The leaves of this plant strewed in granaries are said to destroy the corn-weevil.—*Lightfoot.* It contains so great a quantity of nitre, that in making an extract from it, the mass has taken fire. *Ditto, and Withering.*

12. Urtica. *U. dioica, (U. Urens, improperly.)*

*Ang.* Nettle, common nettle.

*Gen. Desc.* Flowers, male and female apart; calyx 4-leaved; bloss. o. Male, nectary in the centre, glass-shaped. Fem. cal. 2 opposite leafits very small. Summit hairy. Seed 1, egg-shaped, shining. Bloss. o.

*Spec. Desc.* M. and Fem. flowers on distinct plants; *leaves* opposite, heart-shaped; bunches in pairs. *Ditch banks, rubbish, v. comm.* Bloss. July.

*Use.* As a *styptic* this plant was formerly much used, and various hæmorrhagic affections have been mentioned, in which it has been successfully employed. It is said also to manifest a *diuretic* character, and to be useful in calculous complaints, scurvy, gout, jaundice, &c.; but it is now disregarded in medicine, and considered merely as an oleraceous plant, being found, when young, a good substitute for greens and other pot herbs.—*Woodville.* The young shoots in spring are boiled and eaten by the country people instead of cabbage greens.—*Lightfoot.* It was formerly used as an *astringent,* and was considered of service in all kinds of hæmorrhagies, but is now disregarded. A leaf

---

* Three ounces of the juice taken internally, or an external fomentation, have been found useful in the strangury.

leaf put upon the tongue and pressed against the roof of the mouth, is pretty efficacious in stopping a *bleeding at the nose.* In paralytic limbs, and other cases of torpor or lethargy, the fresh leaves have been used in the way of a rubifacient, producing considerable irritation and inflammation, (a practice termed *urtication*) and have been found of advantage in restoring excitement.—*Woodville.* The young shoots are gathered early in spring to boil with broth or gruel. The stalks may be dressed like flax or hemp for making cloth, paper, ropes, nets, &c. (a practice not uncommon in some parts of Russia and Siberia. *Falk.*) The leaves are cut to pieces to mix with the food of young turkeys, and other poultry; and, when a little withered, are eaten by cows.—*Withering.* It is said to be poisonous to frogs, for if a plant be thrown into a vessel where these animals are confined, they soon begin to swell, and in a few days perish.—*Woodville.* In Arran and other islands of Scotland, a rennet is made of a strong decoction of nettles, which, with a quart of salt to three pints, is bottled for use: and of this liquor a common spoonful will coagulate a large bowl of milk very readily and agreeably. The root boiled with alum will dye yarn of a yellow colour.—*Lightfoot.* The stings are very curious microscopic objects; they consist of an exceedingly fine-pointed tapering hollow substance, with a perforation at the point and a bag at the base. When the sting is pressed upon, it readily punctures the skin, and the same pressure forces up from the bag an acrimonious fluid, which instantly enters the wound and excites a burning inflammation, known to the experience of most people. *Hooke Discov. by Micros.* Cows, horses, sheep, goats and swine refuse the leaves; asses are fond of them, and cows will eat them in hay.

13. VISCUM. *V. album.*

*Ang.* Misseltoe. Missel. White Misseltoe.

*Gen. Desc.* M. and Fem. flowers on different plants; bloss. o. M. cal. 4-div.; filaments, o; anthers fixed to the calyx. F. cal. 4 leaves, superior; style, o; berry, pulpy, 1-celled, 1-seeded. Seed, heart shaped.

*Spec. Desc.* *Leaves* spear-shaped, blunt. *Stem* forked. *Spike* axillary. *Flowers* greenish white. *Berries* white. The *root* insinuating its fibres into the woody substance of the tree on which it grows; a singular parasitical evergreen shrub. *On Apple trees chiefly; on many species of forest trees; rarely on oak; in Worcestershire and Herefordshire*

*fordshire very common, rare in the Northern countries; in Ireland unknown.* Bloss. May.

*Use.* The *Viscus quernus* had formerly great reputation for the cure of *Epilepsy*; and a case of that disease in which it proved remarkably successful, is mentioned by Boyle, *Usefulness of Nat. and Exp. Phil.* 174. It was some years afterwards strongly recommended in various *convulsive* disorders by Colbach, who has related several instances of its good effects: *Diss. concerning Misseltoe, &c.* He administered it in substance in doses of ½ drachm or 1 drachm of the wood or leaves; or an infusion of an ounce. This author was followed by others, who have borne testimony to its efficacy, not only in convulsive affections, but also in those complaints denominated *nervous*, in which it is supposed to act as a *tonic*. It has fallen however into general neglect, though it seems to be a remedy well deserving of notice.—*Woodville.* This plant was formerly in great repute as a remedy for *epileptic* and other complaints, but it is now much disregarded. From its berries and bark, bird-lime may be made. The berries are eaten by the Misseltoe-bird, the thrush, &c. and passing through them unchanged, adhere with their excrements to the branches of trees, where they vegetate, being washed by the rain to the underside of the branch, from which invariably their root springs. No art has yet made this plant take root in the earth: but the berries, when fully ripe, will adhere closely, if rubbed on the smooth bark of almost any tree, and produce plants the following winter. Sheep will eat greedily of this plant, and it is said to preserve them from the rot: in hard weather, it is often cut off the trees for them.—*Withering.*

14. Hippophae. *H. rhamnoides.*

*Ang.* Sea buckthorn. Common sallow-thorn.

*Gen. Desc.* M. and F. flowers on different plants. Bloss. o. M. cal. 1-leaf, 2-lobed. Fem. cal. 1 leaf, tubular-berry, superior, 1-celled. Seed, hard, shining.

*Spec. Desc.* *Leaves* strap-spear-shaped, very entire, green above, scaled, white underneath, mid-ribbery prominent. *Flowers* solitary, appear before the leaves. *M. fl.* below the leaf, between a branch and a bud; *Fem. fl.* in the bosom of the lowermost leaves, sitting. *Stem* 8 feet high; *branches* spreading straight, stiff, thorny at the ends. *Sea shore in sand.* Bloss. March—May.

*Use.* Of the berries, which are very acid with an austere vinous taste, the fishermen of the gulph of Bothnia prepare a rob, that added to fresh fish, imparts a very grateful flavor. In sunny sandy situations, it is planted for hedges. *Linnè*. Horses, sheep and goats eat it; cows refuse it.

15. Alchemilla. *A. vulgaris.*

*Ang.* Common Lady's mantle. Bear's-foot.

*Gen. Desc.* Cal. 8-cleft; bloss. 0; seed 1 or 2, inclosed by the calyx.

*Spec. Desc.* *Leaves* gashed, generally seven-lobed, serrated; a rib from the leaf-stalk along the middle of each lobe; *flowers* yellowish green, forming a kind of umbel, the *general involucrum* entirely, the *partial* only half-way surrounding the stalk, *Meadows and pastures.* Blossom June—September.

*Use.* This whole plant is *astringent.* In the province of Smolandia in Gothland, a tincture is made of the leaves, which is given in spasmodic or convulsive diseases. —*Withering.* Horses, sheep, and goats eat it; cows are not fond of it; swine refuse it.

Tetrandria. Digynia.

16. Betula. *B. alba.*

*Ang.* Birch tree. Birk.

*Gen. Desc.* Male and female flower separate, on the same plant; calyx one-leaf, three or five-cleft, blossom four-div. male cal. three-flowered; fem. cal. two-flowered. Seeds 2 or 3.

*Spec. Desc.* A tall tree. *Leaves* triangular spear-shaped, acute, smooth, doubly serrated. *Male catkin,* scales tipped with brown, smaller scales fixed to the centre. *Blossom* egg shaped, concave, green. *Woods, moist hedges.* Bloss. April, May.

*Use.* If a hole be bored in the tree when the sap is rising in spring, a liquor well known for its saccharine qualities distils from it, which, properly fermented with the addition of sugar, makes an agreeable and wholesome wine. The leaves afford a *yellow dye.* The wood is firm, tough, and white; useful to the turner and the cooper; packing boxes and heels for women's shoes are made of it, and the knotty excrescences afford a beautiful varied wood. It affords excellent fuel, and makes the best charcoal; the soot is a good lamp black for making printer's ink. From the north of Lancashire and from Dungarvan, besoms made of its twigs are exported. The bark is extremely useful in the north of Europe; the Highlanders use it to tan tan leather, and make ropes of it; the outer rind they sometimes burn as candles, and it abounds so much with a resinous matter which is highly inflammable, that it is manufactured, sliced, and twisted together into torches; with the fragments interwoven the Swedish fishermen and Laplanders make shoes and baskets. Large thick expanded pieces, with a hole in the middle to fit the neck, they use as a surtout to keep off the rain. The Poles, Swedes, and Russians cover their houses with it in lieu of tiles; and over it the Norwegians lay turf three or four inches thick. The Americans make entire canoes of it. Before the invention of paper, it was used by the ancients for the purpose of writing on. In Kamschatka, hats and drinking cups are made of it. The celebrated *moxa*, or touch wood of the Laplanders, used by them as a cautery in the most acute disorders, is made of the yellow fungous excrescences of the woody part of this tree, which sometimes swell out between the fissures and crevices of it, resembling in substance the agaric. This tree is hurtful to pasturage, but it bears cropping well, and thrives in all kinds of soil, though best in shady places.—*Withering, Lightfoot, &c.* Horses, cows, sheep, and goats eat it; swine refuse it.

17. BETULA. *B. alnus.*

*Ang.* Alder, owler, oller.

*Gen. Desc.* *As above.*

*Spec. Desc.* A tall tree. Leaves roundish, glutinous, clammy, serrated; veins underneath, woolly at the base; *fruit-stalks* branched, wedge-shaped, very blunt; *catkins* brown. *Near water, moist soil.* BLOSS. Feb. March.

*Use.* The whole plant is *astringent*. The fresh gathered leaves, which are covered with a glutinous liquor, some people strew upon their floors to destroy fleas; the fleas are said to be entangled in that tenacious liquor, as birds are in bird-lime. Mr. Pennant says, that about Dundonald in the Highlands of Scotland, the boughs are used as manure; cut in the summer, spread over the fields, and left during the winter to rot; in March the undecayed parts are cleared away and the ground ploughed. The bark dyes a *red* colour, and, with copperas, a *black*. It is also used to dye *brown*, particularly thread, and for colours to be saddened by copperas. It is principally used by the fishermen to stain their nets. The catkins dye *green*. The wood is soft and brittle, but it endures a long time under water, and is therefore used for pipes, and for laying under the foundations of buildings situated on bogs. Turners use it for shoe-heels, ploughman's clogs, and other articles.

Grass grows well beneath the shade of alder; but if this tree be planted in a low meadow, the ground around it will become boggy; whereas round the ash, it becomes firm and dry.—*Withering.* Horses, cows, sheep, and goats eat it; swine refuse it.

18. Betula. *B. nana.*

*Ang.* Dwarf birch tree.

*Gen. Desc.* *As above.*

*Spec. Desc.* *Leaves* circular, scolloped; low shrub, upright. *Trunk* hard, stiff. Bark brown, roughish. Branches expanding, straight, scattered, tapering, woolly, gummy at the base. Catkins half an inch long. *On mountains and wet heaths, bogs.* Bloss. May.

*Use.* The leaves afford a finer *yellow* dye than that of the betula alba, or common birch. This shrub supplies the Laplander, in the summer, when he lives in the mountains, with fuel for the fires that he is obliged constantly to keep in his hut to defend him from the gnats; and, covered with the skin of the rein-deer, it forms his bed.—*Linnè.*

19. Myrica. *M. gale.*

*Ang.* Sweet gale, goule, sweet willow, Dutch myrtle.

*Gen. Desc.* Male and female fl. on different plants, in catkins. Calyx 2 leaves; blossom 0. Female drupa one-celled, superior. Seed 1.

*Spec. Desc.* *Leaves* spear-shaped, convoluted, sprinkled with resinous points, serrated towards the end, on leaf stalks. *Stem* shrub like, smooth rust coloured, with white dots. *Flower-buds* above the leaf-buds at the ends of the branches, which as soon as the fructification is completed, die, the leaf-buds on sides shooting out and the stem becoming compound. *Buds* composed of nine leafy shining scales. *Flowers* appear before the leaves. *Female spike* oblong, five rows, in each five *berries,* which are thickish, roundish, angular, three shallow clefts, to each of which a small tooth is fixed sprinkled with golden resinous dots. *On bogs in gravelly soil, generally in large quantities.* Bloss. May.

*Use.* The leaves have a bitter taste and an agreeable odour; in the Highlands and Hebrides, an infusion or tea made of them, is given to children to destroy *worms.*—*Lightfoot.* The Welch also use it as a *vermifuge* both in powder and infusion, and also applied externally to the abdomen. They lay branches of it under their beds to keep off fleas and moths, and they use it for dying wool yellow.—*Penn.* The Swedes use it, gathered in autumn, to dye dye their yarn yellow, and sometimes employ a strong decoction of it to kill *bugs* and *lice,* and to cure the *itch.*—*Linnè.* It was formerly used by northern nations, and is still in the western isles and Highlands of Scotland, instead of hops; but unless it be boiled a long time, it is apt to occasion head-aches. The cones, (or catkins) boiled in water, throw up a watery scum capable of being made into candles, similar to those made by the Americans from another species of this plant, the *M. cerifera.* It is used to *tan* calf-skins. Its essential oil rises in distillation. Sailors are fond of besoms made of it for sweeping their ships.—*Lightfoot, Withering.* Linnè suspects from the smell, that camphor might be prepared from this plant. Horses and goats eat it; cows and sheep refuse it.

20. CUSCULA. *C. Europæa.* *C. epurtica.* *C. eperica.* *Ang.* Dodder.

*Gen. Desc.* Calyx four or five-cleft; blossom one petal; caps. two-celled, cut round. Seeds in pairs.

*Spec. Desc.* *Flowers* sitting, four-cleft, whitish. *Leaves* 0. A paralytical plant without seed-lobes. *In hops, nettles, heath, &c.* BLOSS. July, August.

*Use.* This is a very curious and singular plant, which will not grow in the ground. *See Bot. Acc. of it.* It was formerly celebrated as a *cathartic,* but is a very languid one, and now out of use.—*Hill.*

Tetandria Tryginia.

21. BUXUS. *B. sempervirens.* *B. aborescens.*

*Ang.* Box.

*Gen. Desc.* M. and fem. fl. on the same, or on different plants. M. calyx three-leaved; bloss. two-pet. germen, a rudiment. Fem. calyx four-leaved; bloss. three-pet. caps. three-celled, three-beaked. Seeds 2.

*Spec. Desc.* M. and fem. fl. on the same plant. *Leaves* oval, thick, glossy. *Blossoms* greenish white or yellow. Fruit a dry capsule. *Woods.* BLOSS. April.

*Use.* An empyreumatic oil, distilled from the shavings of the wood, is often used as a topical application for the *piles,* and seldom fails to procure ease; it will frequently cure the *tooth-ache,* and it has been given internally in *epilepsies.* The leaves powdered destroy *worms.* The wood is very hard, smooth, and a fine yellow, and not being apt to warp is well adapted for the use of the turner; it is made into combs, mathematical instruments, knife handles, button moulds, &c. In the south of Europe it is cultivated in pots, like myrtle with us.—*Withering.*

[ To be continued. ]

